# The In Vivo Granulopoietic Response to Dexamethasone Injection Is Abolished in Perforin-Deficient Mutant Mice and Corrected by Lymphocyte Transfer from Nonsensitized Wild-Type Donors

**DOI:** 10.1155/2015/495430

**Published:** 2015-05-04

**Authors:** Pedro Xavier-Elsas, Cássio Luiz Coutinho Almeida da Silva, Bruno Marques Vieira, Daniela Masid-de-Brito, Túlio Queto, Bianca de Luca, Thiago Soares de Souza Vieira, Maria Ignez C. Gaspar-Elsas

**Affiliations:** ^1^Departamento de Imunologia, Instituto de Microbiologia Paulo de Góes, Universidade Federal do Rio de Janeiro, 21941-590 Rio de Janeiro, RJ, Brazil; ^2^Departamento de Pediatria, Instituto Nacional de Saúde da Mulher, da Criança e do Adolescente Fernandes Figueira, FIOCRUZ, 22250-020 Rio de Janeiro, RJ, Brazil

## Abstract

Exogenously administered glucocorticoids enhance eosinophil and neutrophil granulocyte production from murine bone-marrow. A hematological response dependent on endogenous glucocorticoids underlies bone-marrow eosinophilia induced by trauma or allergic sensitization/challenge. We detected a defect in granulopoiesis in nonsensitized, perforin-deficient mice. In steady-state conditions, perforin- (Pfp-) deficient mice showed significantly decreased bone-marrow and blood eosinophil and neutrophil counts, and colony formation in response to GM-CSF, relative to wild-type controls of comparable age and/or weight. By contrast, peripheral blood or spleen total cell and lymphocyte numbers were not affected by perforin deficiency. Dexamethasone enhanced colony formation by GM-CSF-stimulated progenitors from wild-type controls, but not Pfp mice. Dexamethasone injection increased bone-marrow eosinophil and neutrophil counts in wild-type controls, but not Pfp mice. Because perforin is expressed in effector lymphocytes, we examined whether this defect would be corrected by transferring wild-type lymphocytes into perforin-deficient recipients. Short-term reconstitution of the response to dexamethasone was separately achieved for eosinophils and neutrophils by transfer of distinct populations of splenic lymphocytes from nonsensitized wild-type donors. Transfer of the same amount of splenic lymphocytes from perforin-deficient donors was ineffective. This demonstrates that the perforin-dependent, granulopoietic response to dexamethasone can be restored by transfer of innate lymphocyte subpopulations.

## 1. Introduction

Glucocorticoids have been extensively characterized as being anti-inflammatory [1] and/or immunosuppressive [2] in therapeutic settings and are generally discussed as opposed to the development of inflammation and limiting of production or maturation of some effector immune cell types, in a physiological context [3]. Nevertheless, there is a well-established association between stress and allergic diseases, including asthma [4, 5]. Glucocorticoids, which are an essential part of systemic stress responses, play coadjuvant roles in promoting inflammation and may promote Th2-type immunity through differential effects on Th1 × Th2 cytokine production [5, 6].

Chronically stimulated eosinophil production (eosinopoiesis) is an important feature of human asthma [7] and of murine allergic asthma models [8]. In both cases, allergen challenge of sensitized subjects increases eosinopoiesis in the bone-marrow [7, 8]. This effect is antigen-specific and can be abolished by inducing oral tolerance to the allergen, which affects both eosinophils and neutrophils in bone-marrow [9]. The effects of oral tolerization in bone-marrow neutrophil and eosinophil granulocytes can be duplicated by transfer of splenic T lymphocyte subpopulations from tolerized/sensitized/challenged donors to histocompatible naive recipients [9]. These observations highlight the importance of* acquired* cellular immunity in regulating the hematological response to allergen sensitization and challenge. They further suggest the possibility that granulopoiesis, encompassing both eosinophil and neutrophil production, might be regulated by lymphocyte populations in nonsensitized subjects as well.

In an allergic asthma model, we demonstrated a critical role for endogenous glucocorticoids in the hematological response to allergen challenge: challenge induces a corticosterone surge that is paralleled by increased eosinophilia of bone-marrow in vivo and by increased responsiveness to IL-5, the major eosinopoietic cytokine, ex vivo; the bone-marrow response to challenge is abolished by blockade of glucocorticoid signaling or glucocorticoid production [10]. On the other hand, in the absence of allergen sensitization and challenge, we have also obtained evidence of a link between the corticosterone surge induced by mild surgical trauma and short-term bone-marrow eosinophilia; again, blockade of endogenous glucocorticoid production or action abolished the bone-marrow eosinophilia induced by trauma [11]. These in vivo effects of corticosterone, an endogenous glucocorticoid released by the adrenal glands [10], are paralleled by those of exogenously provided corticosterone or dexamethasone on murine bone-marrow [12, 13] and of other glucocorticoids on human hemopoietic cells [14]. In BALB/c mice, dexamethasone increases eosinophil production in murine bone-marrow culture [12, 13] and primes bone-marrow cells in vivo for increased ex vivo responses to IL-5 [12]. During further screening of inbred mouse strains for differences in the granulopoietic responses to dexamethasone, we observed bone-marrow eosinophilia in mice of the C57BL/6 (B6) background injected with dexamethasone, which was undetectable in perforin-deficient B6 mutants [15] submitted to the same treatment.

Perforin is a major mediator of cellular immunity [15–19], expressed in lymphocyte populations which fight viral [15] and bacterial pathogens [16, 17] as well as malignant cells [18] in the context of both innate and acquired immune responses. Perforin is also expressed by murine bone-marrow neutrophils, which have a critical regulatory role in T cell-mediated contact hypersensitivity [19]. Perforin deficiency is known to induce complex changes in leukocyte populations in humans and mice [16, 17, 20], including a classical presentation of familial hemophagocytic lymphohistiocytosis, characterized by early life onset, high mortality, and multiple immunological defects, including uncontrolled activation and proliferation of CD4+ and CD8+ T cells, cytokine storm, macrophage activation and proliferation, pancytopenia, and anemia [20]. Here we report that perforin deficiency also presents a selective defect in granulocyte production, which can be corrected by wild-type lymphocyte transfer.

## 2. Methods

### 2.1. Reagents

Fetal bovine serum (cat. SH30088.03), RPMI1640 (SH30011.01), and IMDM (SH30228.01) were from Hyclone (Logan, UT, EUA); L-glutamine (G7513), penicillin-streptomycin solution (P4333), essential amino acids solution (50x) (M5550), methylcellulose (M0387), dexamethasone (21-phosphate, disodium salt, D1159), Mifepristone (RU486, M8046), and Histopaque-1083 (10831) were from Sigma-Aldrich Corporation (St. Louis, MO, EUA); Agar Noble (0142-15/21422) was from Difco (Detroit, MI, EUA); nonessential amino acids solution (100x), (11140-050) and MEM vitamin solution (100x) (11120-052) were from GIBCO Life Technologies (Carlsbad, CA, USA); recombinant murine IL-5 (405-ML-025) was from R&D Systems (Minneapolis, MN, USA); recombinant murine GM-CSF (315-03) was from PreproTech (Rocky Hill, NJ, USA); rat antimouse CD8b (Clone: eBioH35-17.2, 12-0083-82, 0.2 mg/mL) was from eBioscience (San Diego, CA, USA); magnetic microspheres conjugated to antimouse CD4 (L3T4, 130-049-20) and to goat antirat IgG (130-048-501, as secondary antibody to primary rat antimouse CD8b) were from Miltenyi Biotec (Ambriex, SP, Brazil).

### 2.2. Animals and Ethical Aspects

Wild-type C57BL/6, perforin-deficient (Pfp) mutants of the B6 background, and wild-type B6.129 mice were bred by CECAL-FIOCRUZ, Rio de Janeiro, Brazil. The Pfp stock was derived from the original B6.129S6-Pfp^tm1Clrk^ stock [15] by backcrossing to C57BL/6 at FIOCRUZ. Unless otherwise indicated, the wild-type controls for the experiments shown were C57BL/6; in selected experiments, B6.129 wild-type controls were used and yielded the same results as C57BL/6 (not shown). The animals were housed and handled following institutionally approved guidelines under License L-00209 from CEUA-FIOCRUZ. Euthanasia was by CO_2_ narcosis. For age and weight of the animals, see Section 3. Routinely, female mice were used for the experiments, since male mice characteristically fight for dominance in the same cage, and the stress associated with fighting may confound the interpretation of the results. We have no evidence, however, that the granulopoietic responses described here, including the strain differences, are restricted to females.

### 2.3. In Vivo and Ex Vivo Procedures

Dexamethasone in saline solution was injected i.p. (200 *μ*L, i.p., amounting to 5 mg/kg; [12]). Controls received saline only. All sample collection was done 24 h later. Where indicated, RU486 in 0.1% methylcellulose was given intragastrically 2 h before dexamethasone with a gavage needle (200 *μ*L, amounting to 100 mg/kg [21]). For lymphocyte transfers, 10^7^ nylon-wool purified cells [22] from naive C57BL/6 donors in a 100 *μ*L volume of sterile saline were injected into the tail vein of Pfp recipients, once. Controls received an equal number of cells from Pfp donors. Where lymphocytes depleted of CD4+ or CD8+ cells were used, the amount of cells injected was that recovered from 10^7^ initial unseparated lymphocytes, in 100 *μ*L sterile saline, i.v. Dexamethasone was administered to the recipients, in both cases, 48 h after lymphocyte transfer. Samples were therefore collected 72 h after transfer. Where indicated, bone-marrow, peripheral blood from the abdominal vena cava, spleens, and whole thymuses were collected, for enumeration of cells or determination of relative weight of the thymus (mg/g body weight) [21, 23]. Bone-marrow collected from both femurs of each mouse in 5 mL RPMI1640 medium/1% FBS with a 22-gauge needle and kept on ice was used for total and differential counts and cell culture (see below). Spleen mononuclear cells were the source for isolation of lymphocytes (see below) [24, 25].

### 2.4. Bone-Marrow Methods

Total cells diluted in Turk's solution were counted in a haemocytometer. Differential counts were carried out in cytocentrifugates after fixation in PBS-10% formaldehyde and staining for eosinophil peroxidase (EPO [26, 27]) followed by counterstaining with Harris' hematoxylin [8]. EPO is a lineage-selective marker detectable at all stages of eosinophil differentiation [27]. Liquid bone-marrow cultures were established at 37°C, with 10^6^ bone-marrow cells in 1 mL RPMI1640/10% FBS, in 5% CO2/95% air, plated in 24-well plates (cat. N°: 142475, NUNC Brand Products) with IL-5 (1 ng/mL; 7 days [8]). Where indicated, dexamethasone was added (10 *μ*L/well, to 10^−7^ M final concentration [12]). Cytocentrifugates were prepared after collection of the cultures and stained for EPO. Absolute numbers were calculated from total cell counts in Turk's solution multiplied by the % of EPO+ cells (eosinophil-lineage cells, both mature and immature) in cytocentrifuge smears. Triplicate* semisolid* (clonal) bone-marrow cultures were established for 7 days with 2 × 10^5^ cells adjusted to 1 mL of 1 : 1 IMDM/agar mix, in 35 mm tissue culture plates (Nunc), in the presence of GM-CSF (2 ng/mL final [12]), with or without dexamethasone (10^−7^ M), at final 20% FBS and 0.3% agar concentrations. Total colonies (defined as aggregates >50 cells derived from a single progenitor cell) [8, 12] were scored at day 7 under an inverted microscope (400x, phase contrast).

### 2.5. Lymphoid Cell Methods

For lymphocyte isolation, spleens were collected and minced in RPMI1640/1% FBS on tissue culture plates. Spleen mononuclear cells (2 × 10^7^ in 10 mL RPMI1640/1% SFB) were isolated by centrifugation on a 1.083-density Ficoll-Hypaque cushion (3 mL, at 400 ×g, for 30 minutes, at room temperature, following manufacturer's instructions) [24, 25]. Cells recovered from the medium/Ficoll-Hypaque interface were collected, washed in serum-free medium, resuspended, counted, and further separated on nylon-wool columns [24, 25] at 4 × 10^8^ cells/2 mL/g nylon wool [24]. Cells eluted in a total 25 mL warm medium, dropwise, were washed and counted before cytocentrifugation/staining or incubation with antibodies. Negative selection [24] was done with MidiMACS immunomagnetic separation device (cat. 130-042-302, Miltenyi Biotec) using LS+ MACS (cat. 130-042-401) columns. Depletion of CD4+ cells was done with 10 *μ*L L3T4/10^7^ nylon-wool purified lymphocytes in 100 *μ*L serum-free RPMI1640, on ice, following manufacturers' instructions. Depletion of CD8+ cells was in two steps, with rat antimouse CD8b conjugated with PE, followed by goat antirat IgG (20 *μ*L/10^7^ lymphocytes in 80 *μ*L medium, on ice). Columns were eluted with 9 mL medium, dropwise, over a 5-minute period. The depleted lymphocyte populations were pelleted and resuspended for transfer like the unseparated lymphocytes.

### 2.6. Statistical Procedures

**Figure 1:**
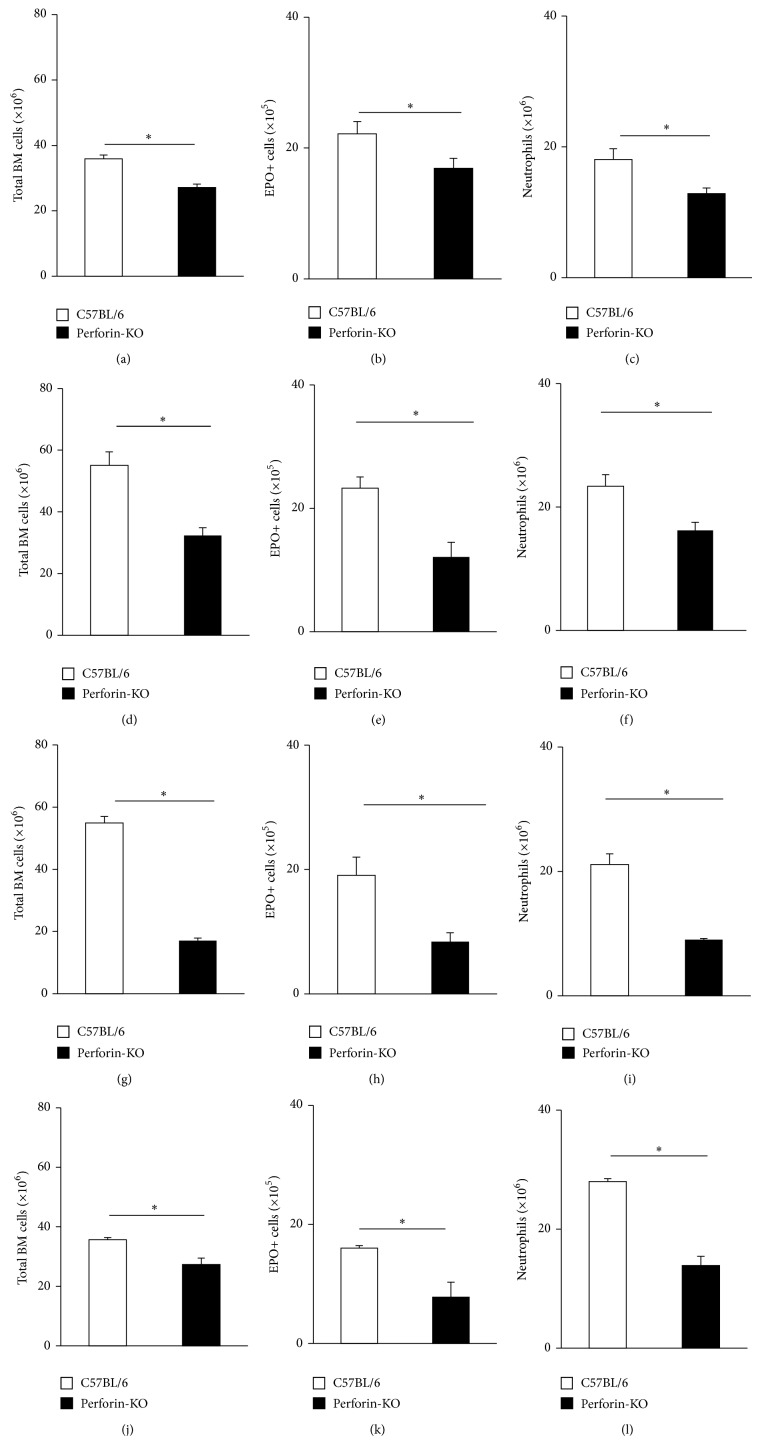
Total and granulocyte counts in freshly harvested bone-marrow of Pfp mice and B6 wild-type controls. Data are Mean + SEM of the counts of total nucleated cells ((a), (d), (g), (j)), EPO+ cells ((b), (e), (h), (k)), and neutrophils ((c), (f), (i), (l)) in C57BL/6 (white bars) and perforin-deficient (Pfp, black bars) bone-marrow. (a)–(c) No matching of age or weight between mutant and control groups; (d)–(f) groups matched by weight (median = 21 g, range 19 g–23 g); (g)–(i) groups matched by age (all at 12 weeks); (j)–(l) groups matched by weight and age. For B6 and Pfp mice, respectively, in (a), *n* = 44 and *n* = 48; in (b), *n* = 27 and *n* = 30; in (c), *n* = 27 and *n* = 24; in (d) and (e), *n* = 6 for both; in (f), *n* = 5 and *n* = 6; in (g), (h), and (i), *n* = 7 for both. In (j), (k), and (l), *n* = 3 for both.  ^∗^Significant differences between the indicated groups.

**Figure 2:**
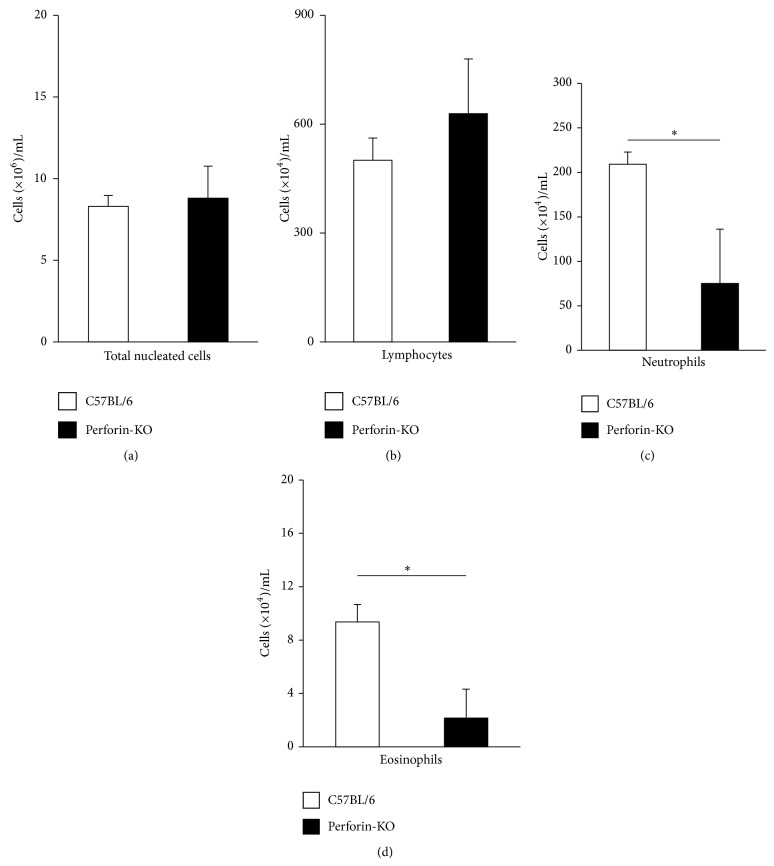
Total leukocyte, lymphocyte, and granulocyte counts in peripheral blood of Pfp mice and B6 wild-type controls. Data are Mean + SEM of total leukocyte (a), lymphocyte (b), neutrophil (c), and eosinophil (d) in 1 mL of peripheral blood of B6 (white bars) and Pfp (black bars) matched by weight (median = 21 g, range 19 g–23 g; *n* = 6 for both).  ^∗^Significant difference between the indicated groups.

**Figure 3:**
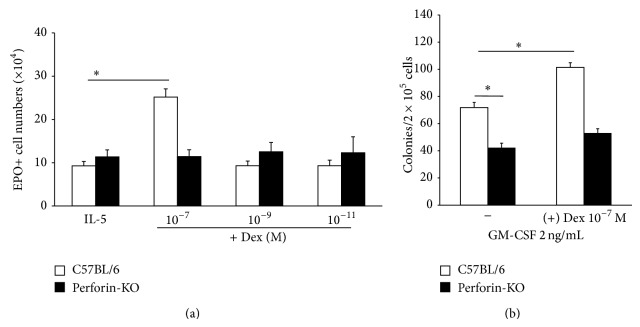
Effectiveness of dexamethasone on in vitro eosinopoiesis in B6 and Pfp bone-marrow cultures. (a) IL-5-induced eosinopoiesis in liquid culture. Data are Mean + SEM of the number of EPO+ cells recovered from 7-day cultures established from B6 ((a), white bars) or Pfp ((a), black bars) in the presence of IL-5 (1 ng/mL). Dex, dexamethasone. Data were from 3 separate experiments, carried out in 3 different days, each providing data from 3 B6 and 3 Pfp mice (*n* = 9 for both strains). (b) GM-CSF-stimulated colony formation in semisolid culture. Data are Mean + SEM of colony counts in 7-day semisolid cultures from B6 (white bars) and Pfp (black bars) bone-marrow established in the presence of GM-CSF (2 ng/mL), alone or in association with Dex (10^−7^ M). For cultures without (left) and with (right) Dex, data are, respectively, from B6, *n* = 14 and *n* = 7; Pfp, *n* = 12 and *n* = 6.  ^∗^Significant difference between the indicated groups.

**Figure 4:**
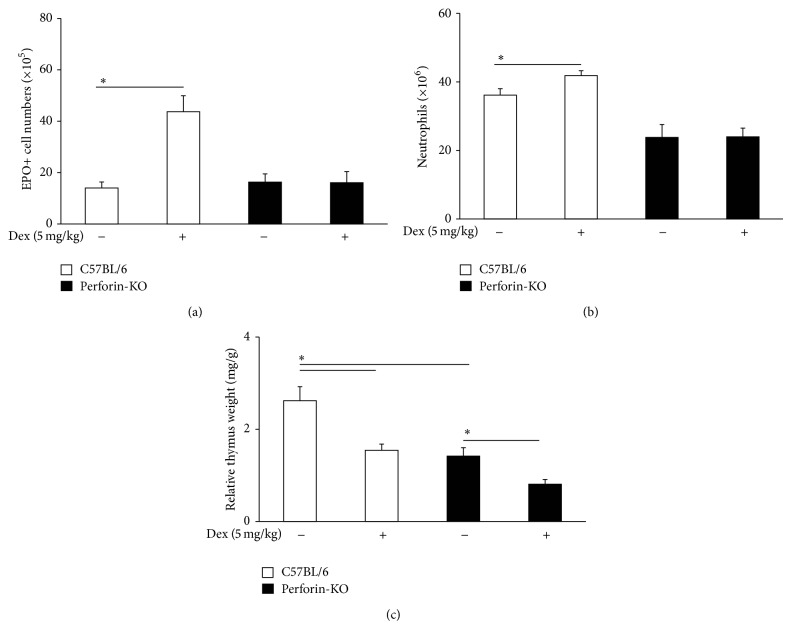
In vivo granulopoietic response to dexamethasone injection in B6 and Pfp mice ((a), (b)). Data are Mean + SEM of the numbers of EPO+ cells (a) and neutrophils (b) in freshly harvested bone-marrow of age-matched (12 weeks) B6 (white bars) or Pfp (black bars) mice, 24 h after i.p. injection of dexamethasone (Dex, 5 mg/kg) or saline. For B6 and Pfp mice, *n* = 3 and *n* = 5, respectively. (c) Data are Mean + SEM of the relative weight of the thymus (mg/g body weight) from age-matched B6 (white bars) or Pfp (black bars) mice, 24 h after injection of dexamethasone or saline as above. For B6 and Pfp mice, *n* = 4 and *n* = 5, respectively.  ^∗^Significant differences relative to the indicated controls.

**Figure 5:**
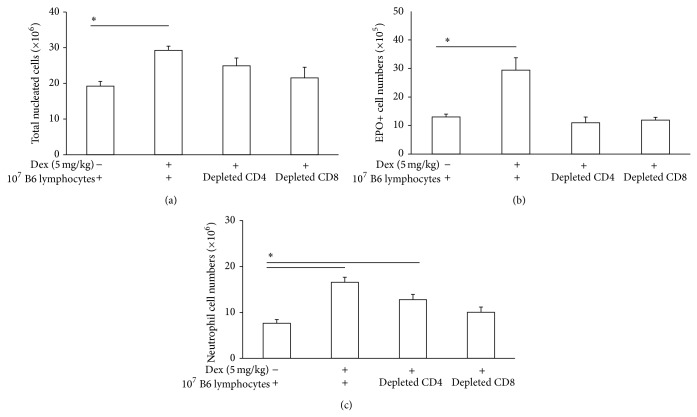
Short-term reconstitution of responses to dexamethasone in Pfp mice by transfer of B6 splenic lymphocytes. Data are Mean + SEM of the total nucleated cells (a), EPO+ cells (b), and neutrophils (c) in freshly harvested bone-marrow of Pfp mice which had received total nylon-wool purified wild-type lymphocytes, or CD4-depleted lymphocytes, or CD8-depleted lymphocytes, i.v., 48 h before dexamethasone (5 mg/kg) i.p. injection. For controls given total lymphocytes before saline i.p. (first column, from left to right) and for experimental groups given total lymphocytes (second column), CD4-depleted lymphocytes (third column), or CD8-depleted lymphocytes (fourth column) before dexamethasone, groups had, respectively, *n* = 4, *n* = 10, *n* = 7, and *n* = 4.  ^∗^Significant differences between the indicated groups.

**Figure 6:**
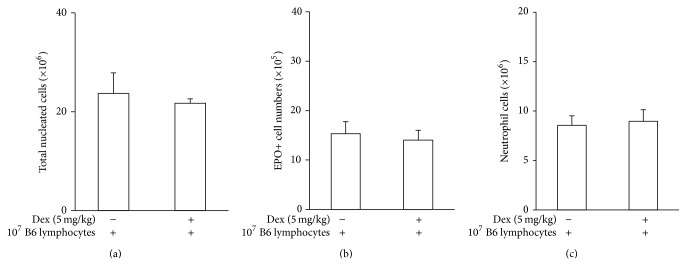
The transfer of splenic lymphocytes from a Pfp donor to a Pfp recipient fails to restore responses to dexamethasone. Data are Mean + SEM of total nucleated cell (a), EPO+ cell (b), and neutrophil (c) counts in freshly harvested bone-marrow of Pfp mice which had received 10^7^ total nylon-wool purified Pfp lymphocytes, i.v., 48 h before dexamethasone (5 mg/kg) injection. For both saline-injected and dexamethasone injected mice, *n* = 3.

Data are presented as Mean + SEM. The numbers of experiments (*n*) are indicated in the caption of the figures, to avoid overcharging the figures and captions. For comparisons of two groups (Figures 1 and 2), we used the two-tailed *t*-test with separate variances (Systat for Windows, version 5, Systat Inc., Evanston, IL). For multiple comparisons (Figures 3, 4, 5, and 6), we used ANOVA, with the Tukey HSD correction for groups of equal size (Systat for Windows) or with Bonferroni's correction for groups of unequal size (using Prisma 5 for Windows, Graph Pad, La Jolla, CA), unless otherwise indicated in Section 3.

## 3. Results

### 3.1. Evidence for a Defect in Steady-State Granulopoiesis in Perforin-Deficient Mice

Following preliminary experiments (not shown) that evidenced a positive response to dexamethasone in B6 wild-type mice, as well as the absence of any response, positive or negative, in Pfp mutants of the B6 background, we reviewed data on freshly harvested bone-marrow from a large number of mice of both strains (*n* = 44 and *n* = 48, resp.), to look for evidence of strain differences in bone-marrow steady-state parameters, in the absence of dexamethasone exposure (Figure 1).

A significant difference was observed in this large series, with Pfp mice having lower bone-marrow cellularity than wild-type controls (Figure 1(a); *p* < 0.001). The data available for EPO+ cells (Figure 1(b);  *p* = 0.033) and neutrophils (Figure 1(c); *p* = 0.010) of the mice in the large series showed significant differences as well, with lower counts in Pfp mutants. As the two groups were defined only on the basis of genetic differences (presence or absence of functional perforin genes), these differences could still be accounted for, in principle, by variance due to nongenetic factors within these groups, especially those which influence growth and development. Because bone-marrow cellularity is roughly proportional to the size of the animal, we next evaluated whether these differences would disappear in the comparison between groups of control and mutant mice matched by weight (Figures 1(d)–1(f)). Significant differences were still observed for total cells (Figure 1(d); *p* = 0.001), eosinophils (Figure 1(e); *p* = 0.004), and neutrophils (Figure 1(f); *p* = 0.011), all three parameters being lower in the mutant mice. Because age might affect bone-marrow function through mechanisms unrelated to body weight gain (as senescence may have an earlier onset in some strains; furthermore, age is a major determinant of incidence of many pathological processes, such as malignancies), we next examined whether matching by age (all animals at 12 weeks) would eliminate the differences. Significant differences were still found for all three parameters (for Figures 1(g)–1(i), resp., *p* = 0.001, *p* = 0.006, *p* = 0.001), which were lower in the mutant mice. Even matching by both weight and age (for Figures 1(j)–1(l), resp., *p* = 0.022, *p* = 0.032, *p* = 0.001) failed to eliminate these significant differences between controls and mutants for any of the three parameters, which were all lower in the mutant mice. Overall, this suggests that bone-marrow cellularity, as well as bone-marrow eosinophil and neutrophil counts, is significantly lower in Pfp mutants than in B6 mice and that this difference cannot be dismissed as created by undue comparisons of two groups differing in body weight, age, or both.

We further examined (Figure 2) the counts of total cells, lymphocytes, neutrophils, and eosinophils in peripheral blood of weight-matched (median 21 g, range 19–23 g) B6 and Pfp mice. Unlike bone-marrow, peripheral blood total nucleated cell counts did not differ significantly between these strains (Figure 2(a), *p* = 0.795), nor did lymphocyte counts (Figure 2(b), *p* = 0.417). By contrast, both neutrophil (Figure 2(c), *p* = 0.025) and eosinophil (Figure 2(d); *p* = 0.030) counts were significantly different, and, like in bone-marrow, lower in Pfp mice. Together, the data in Figures 1 and 2 suggest that, even in the absence of dexamethasone, Pfp mice have reduced granulocyte numbers both inside and outside of bone-marrow, relative to wild-type controls of comparable body weight.

### 3.2. In Vitro Response to Dexamethasone in B6 and Pfp Bone-Marrow

Our original observation of this strain difference was increased % eosinophils in cultured bone-marrow from B6, but not Pfp, mice when both IL-5 and dexamethasone were present (Jones and Cardoso de Mendonça, unpublished observations). This is, however, insufficient to characterize a strain difference in response to dexamethasone, because the % of a given cell type in a bone-marrow sample, which has a highly heterogeneous composition, can be increased artifactually by a corresponding decrease in another cell type, rather than by a positive effect of dexamethasone on the cell type of interest. To confirm a positive effect of IL-5 and dexamethasone in stimulation of wild-type and mutant eosinopoiesis, liquid cultures were established from the same number of bone-marrow cells (10^6^) from B6 or Pfp donors, in the presence of IL-5, alone or associated with dexamethasone, and the absolute numbers of eosinophils in the culture were determined. As shown in Figure 3, eosinophils were produced in cultures of IL-5-stimulated wild-type and mutant bone-marrow (Figure 3(a)). Control cultures lacking IL-5 do not contain eosinophils at the end of the culture period (not shown; [8]). Counts of EPO+ cells recovered at the end of the culture were increased in the presence of dexamethasone (10^−7^ M), relative to IL-5 controls, in cultures from B6 controls, but not from Pfp mutants (Figure 3(a), *p* < 0.001). Nevertheless, the IL-5 present was sufficient to sustain eosinopoiesis in the absence of dexamethasone by Pfp bone-marrow to the same level observed in B6 control cultures. On the other hand, data from semisolid cultures, which allowed us to examine the effects of dexamethasone on several classes of progenitors (colony-forming cells), including the granulocyte (G) and granulocyte-macrophage (GM) progenitors, which produce neutrophils, are also shown in Figure 3. The total counts of colonies formed by GM-CSF-stimulated Pfp bone-marrow progenitors, in the absence of dexamethasone, also differed significantly from those of B6 controls (*p* ≤ 0.001): even though identical numbers of bone-marrow cells were plated, less colonies were made by perforin-deficient bone-marrow (Figure 3(b)). Furthermore, dexamethasone (10^−7^ M) significantly stimulated colony formation by B6 bone-marrow (*p* = 0.002) but failed to stimulate (*p* = 0.558) Pfp bone-marrow in the same conditions (Figure 3(b)). These observations show that bone-marrow progenitors from Pfp mice differ significantly from those of B6 controls, because they form less colonies and do not respond to dexamethasone, which significantly enhances colony formation in the wild-type cultures.

### 3.3. In Vivo Bone-Marrow Response to Dexamethasone in Control and Perforin-Deficient Bone-Marrow

We next examined the effect of dexamethasone (5 mg/kg injection [12]) on bone-marrow eosinophils and neutrophils, as well as on the relative weight of the thymus, which is significantly reduced by glucocorticoids [21]. As shown in Figure 4, dexamethasone injection increased the numbers of bone-marrow eosinophils (Figure 4(a), *p* = 0.003) and neutrophils (Figure 4(b), *p* = 0.019) in vivo, relative to saline-injected controls, in wild-type B6 mice, but not in Pfp mice. The effect of dexamethasone on eosinophil numbers in wild-type B6 mice was abolished by RU486 pretreatment, while RU486 had no effect of its own in the absence of dexamethasone, in either B6 or Pfp mice (not shown). The relative weight of the thymus was significantly different (*p* = 0.010) between body weight-matched Pfp mice and wild-type controls, suggesting a decreased thymic cellularity in mutant thymus, even without exogenous glucocorticoid administration (Figure 4(c)). Importantly, dexamethasone injection did reduce significantly the relative weight of the thymus in both wild-type (*p* = 0.007) and mutant (*p* = 0.001) mice (Figure 4(c)). Again, the effect of dexamethasone on the thymus was abolished in both cases by RU486 pretreatment (not shown). Together, these observations show that Pfp bone-marrow is unresponsive to dexamethasone stimulation in vivo, although dexamethasone significantly increases eosinophil and neutrophil numbers in the bone-marrow of wild-type controls. They also show that lack of responsiveness to dexamethasone in Pfp bone-marrow is not due to a general lack of response to glucocorticoids, because the thymus of Pfp mice responds to dexamethasone injection as expected.

### 3.4. Correction of the Defective Response to Dexamethasone in Pfp Mice by Wild-Type Lymphocyte Transfer

Because perforin is mainly expressed in various effector/regulatory lymphocyte subsets, it was important to test whether the perforin-related defect in bone-marrow response to dexamethasone could be corrected by introducing B6 lymphocytes in Pfp mice. We have done so with splenic lymphocytes from naive B6 donors, because both the baseline granulopoiesis defect and the defective granulopoietic response to dexamethasone were observed in the absence of allergic sensitization. The number (10^7^) of lymphocytes for transfer into individual mice was defined on the basis of similar reconstitution studies [23]. To define whether elimination of a particular lymphocyte subpopulation defined by standard surface markers (CD4/CD8) prevented reconstitution of the dexamethasone response, 10^7^ total lymphocytes purified from spleen were submitted to alternative depletion protocols with marker-specific microbeads, and the depleted cells in the column effluent, corresponding to the number of cells negative for the selection marker present in the original lymphocyte sample, were injected.

As shown in Figure 5, Pfp recipients of wild-type lymphocytes (unseparated) show a significant in vivo response to dexamethasone injection, by increased total cell (Figure 5(a); *p* = 0.032, *t*-test), eosinophil (Figure 5(b); *p* = 0.04), and neutrophil (Figure 5(c); *p* < 0.001) counts in the bone-marrow, sharply contrasting with observations in Pfp mice in the absence of wild-type cell transfer (compare Figure 4). On the other hand, depletion of CD4+ or CD8+ cells in the wild-type lymphocyte preparation gave distinct results in the reconstitution assay, depending on the granulocyte population examined: while depletion of either subset abolished the ability of splenic lymphocytes to reconstitute the eosinopoietic response to dexamethasone (Figure 5(b)), depletion of CD4+ cells did not prevent reconstitution of the neutropoietic response (Figure 5(c); *p* = 0.013), while depletion of CD8+ cells prevented reconstitution.

As a further control, we performed transfer of Pfp lymphocytes into Pfp recipients and obtained no reconstitution of dexamethasone responses, judged by any of these three parameters (Figure 6). In these controls, dexamethasone reduced significantly the relative weight of the thymus, showing that dexamethasone was able to reach systemically active levels even if bone-marrow showed no evidence of responding to it.

Overall, these observations suggest that lymphocytes from the spleen of naive wild-type mice, but not Pfp mice, are able to reconstitute in the short term the granulopoietic responses to dexamethasone, but different cells may be involved in reconstitution of lineage-specific (eosinophilic versus neutrophilic) responses.

## 4. Discussion

This is, to our knowledge [15–20], the first description of a selective defect in granulopoiesis associated with perforin deficiency in mice and of its correction by the intravenous transfer of wild-type lymphocytes. As such, it extends the range of manifestations associated with perforin deficiency and raises the issue of how perforin contributes to regulation of granulocyte lineages in vivo. Because the wild-type lymphocyte populations interact with a drug (dexamethasone) which initiates signaling through the RU486-inhibitable glucocorticoid receptor, to stimulate in vivo eosinophil and neutrophil production, these observations may be relevant to processes in which bone-marrow is stimulated by immune responses [9] or trauma [10] with involvement of the same receptor and of endogenous glucocorticoids released by the adrenal glands.

Perforin is a well-characterized effector protein, mainly (but not exclusively) expressed in lymphocytes which share the ability to induce cell-mediated cytotoxicity [15–20]. Perforin deficiency was initially characterized by the loss of important cytotoxic lymphocyte functions [15–20]. However, it was soon realized that perforin deficiency entails other, more complex, functional consequences, leading to the development of type II familial hemophagocytic lymphohistiocytosis [16, 17, 20], which shares pathophysiological features with the macrophage activation syndrome, including interferon- (IFN-) *γ* overproduction. Hence, perforin deficiency has broader consequences in addition to impairment of cytotoxic lymphocyte function, and IFN-*γ*, alone or in association with other “cytokine storm” components, may transduce its effects on other leukocyte lineages.

One of the issues raised by our findings is whether the lower granulocyte numbers in blood and bone-marrow of Pfp mice are due to the progression of the murine equivalent of the human familial hemophagocytic lymphohistiocytosis [16, 17, 20, 28]. It should be noted that the human familial hemophagocytic lymphohistiocytosis is a severe condition, presenting early in life in most cases and associated with important mortality [20, 28]. Also, phenotypically distinct forms correspond to different mutations with perforin expressed at lower levels and/or with altered properties, a situation that is not applicable to the present study, which employed perforin null mice [15]. The perforin-deficient mice in the original Walsh et al. study (from which the mice used in this study descend) were described as healthy for at least 5 months [15]. In our study, mice were certified SPF and maintained in microisolator units [15], with no evidence of mortality, stunting, or signs of infection during the entire observation period of up to 3 months (12 weeks).

On the other hand, Pfp defect in response to dexamethasone could be corrected by transfer of as few as 10^7^ lymphocytes 48 h before the stimulus. The prompt reconstitution of this defect argues against the lack of dexamethasone response in Pfp animals being caused by a chronic inflammatory disease of the bone-marrow, such as familial hemophagocytic lymphohistiocytosis, which in humans is challenging for therapy [28]. Because correction was achieved by simple transfer of lymphocytes from naive donors, we think it is more likely that the transferred lymphocytes provided something that was lacking in the recipient.

Neutrophils were reduced in bone-marrow and blood of Pfp mice relative to B6 controls. Lymphocytes, however, were not. This not only shows that the neutrophil deficiency has peripheral expression but further argues against reduced numbers in bone-marrow being due to an increased export of neutrophils to the periphery. The evidence from colony-forming assays suggests, instead, that production is greatly reduced in Pfp relative to B6 mice, as there were less GM-CSF-responsive progenitors in an identical number of bone-marrow cells. Reduced production would explain the decrease in granulocyte counts both outside and inside of bone-marrow. On the other hand, normal numbers of lymphocytes and total leukocyte counts show that the defect is selective for granulocytes, which are not the major circulating leukocyte subpopulation in mice, so that reduction in their numbers does not have a major impact on total circulating leukocyte counts. Neutropenia is associated with increased susceptibility to bacterial and fungal infection, provided it is severe enough. This was not observed in our study, so presumably the Pfp mice were capable of coping with the microorganisms present in a rather clean environment (SPF conditions, microisolator housing). It remains to be seen, however, whether following a more severe infectious exposure, such as that associated with sepsis induction, Pfp mice would prove more vulnerable to bacterial dissemination. This will be addressed in future studies, since emergency granulopoiesis, as opposed to baseline granulopoiesis, is driven by GM-CSF and related hemopoietic cytokines [29]. The lack of appropriate response to GM-CSF, both in the absence and in the presence of dexamethasone, would predict that emergency granulopoiesis would be defective in Pfp mice and might therefore negatively influence the outcome of sepsis. The hypothalamus-pituitary-adrenal axis is activated in sepsis [30], and therefore it is possible that emergency granulopoiesis is driven by endogenous glucocorticoids, in a way consistent with our observations. The prediction, in this case, is that perforin would be required for normal host response to bacterial sepsis through an effect on glucocorticoid-mediated signaling in the bone-marrow.

The ability of lymphocyte preparations to reconstitute responses to dexamethasone was abolished by depletion of CD4+ and/or CD8+ cells before transfer, depending on the granulocyte population. For neutrophils, CD8+ alone seemed to be required. By contrast, for eosinophils both CD4+ and CD8+ cells were indispensable. While this suggests that different mechanisms are involved for eosinophils and neutrophils, it certainly raises the issue of the lymphocyte subpopulation involved. Since naive lymphocytes are sufficient, it is not likely that conventional T cells, CD4+ or CD8+, are responsible, for they would not be activated in this isolation protocol [24, 25, 31]. Conventional NK cells would not be a strong candidate, since CD4+ or CD8+ lymphocyte depletion would probably not eliminate them [32, 33]. Innate lymphocytes other than conventional NK cells, however, would be expected to be present in naive mouse spleens [32, 33]. Natural killer T cells, which include subsets expressing CD4 and CD8, are found in significant numbers in B6 spleen, present an activated memory phenotype without known activating exposure, and exert multiple regulatory functions through rapid secretion of large amounts of cytokines [32, 33], might perhaps account for some of our observations.

Regardless of innate or adaptive lymphocyte population involved, two questions which require further study are as follows: (a) is perforin the missing component that is brought by the transferred lymphocytes? and (b) do these lymphocytes act by homing to the recipients' bone-marrow and interacting locally with granulocyte progenitors/precursors? Both questions, however, require a more complex experimental design and resources to allow us to detect and modify perforin expression inside living lymphocytes, as well as monitor their homing to the bone-marrow compartment. For this reason, such experiments fall outside the scope of this paper, which is limited to the demonstration of the defect and of its correction.

Finally, it should be noted that lymphocyte transfer restored the short-term response to dexamethasone but did not bring the baseline counts of eosinophils and neutrophils to the same level of the B6 controls. This might be interpreted as evidence that steady-state granulopoiesis and response to dexamethasone are unrelated processes (i.e., their perforin deficiency entails two distinct defects in granulopoiesis, rather than one defect with distinct manifestations). We do not support this interpretation, however, because the duration of the experiment might be insufficient for an effect of lymphocyte transfer alone on steady-state granulocyte numbers to become noticeable. It is also possible that demonstration of a durable effect on steady-state granulocyte numbers would require not only an extended observation period but also an increased or repeated input of wild-type lymphocytes and an interaction of exogenous lymphocytes with endogenous adrenal glucocorticoids as well.
